# Plasma circulating tumor DNA unveils the efficacy of PD-1 inhibitors and chemotherapy in advanced gastric cancer

**DOI:** 10.1038/s41598-024-63486-x

**Published:** 2024-06-18

**Authors:** Rongqi Jiang, Xu Cheng, Ping Li, Enqing Meng, Xinyi Wu, Hao Wu

**Affiliations:** 1 Department of Oncology, The First Affiliated Hospital of Nanjing Medical University, 300 Guangzhou Road, Nanjing, 210029 Jiangsu Province People’s Republic of China; 2 Gastric Cancer Center, The First Affiliated Hospital of Nanjing Medical University, 300 Guangzhou Road, Nanjing, 210029 Jiangsu Province People’s Republic of China; 3 Institute for Gastric Cancer Research, Nanjing Medical University, Nanjing, 211166 Jiangsu Province People’s Republic of China

**Keywords:** Circulating tumor DNA, PD-1 inhibitors, First line treatment, Advanced gastric cancer, ctDNA response, Cancer, Gastrointestinal cancer, Tumour biomarkers, Tumour immunology

## Abstract

Programmed Death Receptor 1 (PD-1) inhibitors, when combined with chemotherapy, have exhibited notable effectiveness in enhancing the survival outcomes of patients afflicted with advanced gastric cancer. However, it is important to acknowledge that not all patients derive substantial benefits from this therapeutic approach, highlighting the crucial necessity of identifying efficacious biomarkers to inform immunotherapy interventions. In this study, we sought to investigate the predictive utility of circulating tumor DNA (ctDNA) as a biomarker in a cohort of 30 patients diagnosed with advanced gastric cancer, all of whom underwent first-line treatment involving PD-1 inhibitor administration alongside chemotherapy. We procured peripheral blood samples both at baseline and following the completion of two treatment cycles. Additionally, baseline tissue specimens were collected for the purpose of genomic alteration assessment, employing both 47-gene and 737-gene next-generation sequencing panels for plasma and tumor tissue, respectively. We delineated a ctDNA response as the eradication of maximum variant allele frequencies relative to baseline levels. Notably, the objective response rate among individuals exhibiting a ctDNA response proved significantly superior in comparison to non-responders (*P* = 0.0073). Furthermore, patients who manifested a ctDNA response experienced markedly prolonged progression-free survival (PFS) and overall survival (OS) when juxtaposed with those devoid of a ctDNA response (median PFS: 15.6 vs. 6.0 months, *P* = 0.003; median OS: not reached [NR] vs. 9.0 months, *P* = 0.011). In summation, patients with advanced gastric cancer receiving first-line treatment with PD-1 inhibitors and chemotherapy, dynamic changes in ctDNA can serve as a potential biomarker for predicting treatment efficacy and long-term outcomes.

## Introduction

Gastric cancer currently stands as the fifth most prevalent malignancy worldwide and ranks as the fourth leading cause of cancer-related mortality^1^. Over the past decade, the treatment landscape for gastric cancer has undergone significant shifts, primarily due to breakthroughs in immunotherapy and targeted therapy. Clinical data suggest that combining PD-1 inhibitors with chemotherapy significantly prolongs survival in patients with advanced gastric cancer compared to traditional chemotherapy regime^2–4^. Currently, identifying patients likely to benefit from immune checkpoint inhibitor therapy through biomarker screening is crucial for optimizing gastric cancer immunotherapy. Biomarkers such as programmed cell death-ligand 1 combined positive score (PD-L1 CPS)^5^, microsatellite instability (MSI) ^6^, and tumor mutational burden (TMB)^7^, approved by the Food and Drug Administration (FDA) for predicting immunotherapy outcomes, have been validated in multiple clinical studies. The predictive efficacy of biomarkers like Epstein-Barr virus (EBV) infection, POLE/POLD1 mutations, tumor-related ctDNA, and the gastric inflammatory prognostic index (GIPI) in the field of gastric cancer is emerging^8–11^, yet their correlation with immunotherapy requires further research confirmation. Thus, exploring new biomarkers to differentiate between responders and non-responders to immunotherapy is vital for guiding treatment decisions and can help reduce unnecessary side effects and medical expenses.

In exploring biomarkers, we find that gastric cancer exhibits significant spatial and temporal heterogeneity. These complex molecular characteristics not only promote acquired resistance to existing treatments but are also key drivers of disease progression^12^. In clinical practice, tracking molecular changes during the treatment of malignant tumors is crucial for understanding resistance mechanisms and treatment responses. However, the invasiveness of tissue biopsy limits its utility for repeated assessments, and the genomic profile and molecular characteristics of metastatic tumor tissues sometimes differ from the primary tumor^13^, making it challenging to monitor cancer progression at the tissue level. Circulating tumor DNA (ctDNA), a fragmentary form of free cellular DNA originating from the tumor, accumulates in the blood (especially plasma) of cancer patients can provide insights into the tumor genome, although its comprehensiveness is influenced by several technical and biological challenges. Analyzing ctDNA in plasma using next-generation sequencing (NGS) can address the limitations of tissue evaluation. Moreover, compared to tissue biopsy, ctDNA analysis offers the advantages of being non-invasive, cost-effective, and highly repeatable. Currently, ctDNA has shown great potential in identifying genomic changes, determining resistance to targeted therapies, and monitoring tumor relapse or progression^14,15^.

Studies indicate that dynamic changes in ctDNA can effectively predict the response of patients with non-small cell lung cancer^16^ and melanoma^17^ to immunotherapy. Similarly, research on gastric cancer demonstrates that changes in ctDNA levels are closely related to treatment outcomes, with reductions in ctDNA levels often indicating a better prognosis^18,19^. Although these findings theoretically position dynamic ctDNA changes as predictive markers of gastric cancer immunotherapy response, the data primarily come from patients who received immune checkpoint inhibitor therapy after first-line chemotherapy failure or from patients who underwent multi-line immunotherapy without standard immunotherapy protocols. Therefore, further validation is required to confirm the effectiveness of early ctDNA dynamics in accurately predicting the response of patients with advanced gastric cancer to standard first-line PD-1 monoclonal antibody therapy combined with chemotherapy. This study aims to analyze the early dynamic changes in ctDNA during treatment and explore its potential as a biomarker for predicting the efficacy of first-line immunotherapy combined with chemotherapy in patients with advanced gastric cancer.

## Materials and methods

### Study design and patients

Our study encompassed 30 patients diagnosed with advanced gastric cancer, who participated in an biomarker study registered at Chinese Clinical Trial Registry (Chictr.org.cn) (Registration Date: 03/11/2022; Registration number: ChiCTR2200065366). This study received approval from the Review Board of the First Affiliated Hospital of Nanjing Medical University (Registration Date: 26/07/2022; Approval number: 2022-SR-428), and all patients provided informed consent. This study will adhere to relevant regulations, including the Declaration of Helsinki by the World Medical Association, and the entire methodology and procedures will be strictly conducted in accordance with guidelines set forth by the Institutional Review Board.

This research was conducted at the First Affiliated Hospital of Nanjing Medical University in China from July 2022 to June 2023. The inclusion criteria for patient enrollment were as follows: histopathologically confirmed unresectable, recurrent, or metastatic adenocarcinoma of the gastric or gastroesophageal junction, with at least one lesion that met the criteria of being either measurable or non-measurable but evaluable according to RECIST v1.1^20^; the absence of significant cardiac, liver, kidney, or psychiatric conditions; and an ECOG performance status (PS) score of 0/1 within one week prior to the initiation of baseline treatment. Conversely, the exclusion criteria entailed the presence of any other active malignancy and prior exposure to immune checkpoint antibodies or agents, such as PD-1, PD-L1, cytotoxic T lymphocyte-associated protein 4 (CTLA4) inhibitors, and the like.

All patients enrolled in this study were required to complete a minimum of two cycle of combined PD-1 monoclonal antibody and chemotherapy. The initial radiological assessment took place upon the conclusion of two treatment cycles. Subsequently, efficacy evaluations were performed after every two cycles. Following the completion of six treatment cycles, further efficacy evaluations occurred every three months until either disease progression was detected or the most recent follow-up was censored. Blood samples were prospectively collected from each patient at baseline and during efficacy assessments. Only patients with a minimum of two blood samples, including the baseline sample, were eligible for subsequent analysis.

### Blood sample processing and tissue processing

Whole blood samples underwent centrifugation at 4 °C for 10 min at 1600 g. Following this initial centrifugation, the plasma supernatant underwent an additional 10-min centrifugation at 4 °C, with a force of 16,000 g, to eliminate any residual cells and debris. Formalin-fixed paraffin-embedded (FFPE) tissue sections were assessed for tumor cell content using hematoxylin and eosin (H&E) staining. Only samples with a tumor content of ≥ 20% were deemed eligible for subsequent analyses.

### Tumor tissue mutational analysis and ctDNA detection

Tumor tissues underwent analysis for somatic mutations through targeted NGS of 737 cancer-related genes, as previously described^21^. The median de-duped sequencing depths for tumor tissue and peripheral blood leukocytes were 1117× and 572×, respectively. Plasma samples were analysed using a 47-gene NGS panel, achieving a median de-duped sequencing depth of 60,455×, following methods previously published^22–24^. The raw data generated by sequencing is first subjected to quality control analysis using FastQC to ensure it meets the quality standards required for subsequent analyses. Then, the data are aligned to the human reference genome hg19 using Burrows-Wheeler Aligner, generating alignment files. Internally developed software integrates dual UMI sequences at the ends of DNA fragments to create double-consensus sequences, enhancing specificity—especially for low allele frequency variants in cfDNA—using a binomial test-based site-specific variant detection model. Variants are filtered through support counting, strand bias status, base quality, and mapping quality. Additionally, variant identification is optimized for detecting variations in short tandem repeat regions. Single nucleotide variations (SNVs) and insertions/deletions (indels) are annotated using ANNOVAR, retaining only missense mutations, nonsense mutations, frameshift mutations, and non-frameshift insertions/deletions. Copy number variations (CNVs) and gene rearrangements are detected using internally developed software^21^. The tissue and blood panels used in our study can be found in Supplementary Tables 6 and 7.

### ctDNA analysis

For each sample, the maximum and mean Variant Allele Frequency (VAF) were computed using SNVs, Indels, and fusion events extracted from plasma. The maxVAF was defined as the highest allele frequency among the detected somatic variants in each sample. This measure highlights the dominant tumor clone present in the bloodstream at a given time. The meanVAF was defined as the average frequency of all allele frequencies detected in each sample. This metric provides a broader view of the tumor heterogeneity and the overall burden of tumor DNA in the blood. MaxVAF helps in pinpointing the most aggressive tumor components, while meanVAF provides a summary measure of the tumor’s genetic diversity and load. A VAF ≥ 0.3% was deemed as ctDNA detectable. Patients lacking detectable mutations in plasma or harbouring variants with VAF below 0.3% were classified as ctDNA undetectable. In cases where more than one somatic mutation was identified in a baseline plasma sample, the maxVAF was utilised to monitor ctDNA levels over time concerning the baseline. The complete clearance of baseline maxVAF is defined as “ctDNA response”.

### Clinical outcomes

Radiological responses to the combination of PD-1 monoclonal antibody and chemotherapy in patients were assessed and categorized as complete response (CR), partial response (PR), stable disease (SD), or disease progression (PD), following the RECIST v1.1 criteria. Objective Response Rate (ORR) denoted the proportion of patients achieving CR and PR, while Disease Control Rate (DCR) encompassed the proportion achieving CR, PR, and SD. Overall Survival (OS) was calculated from the date of the first PD-1 monoclonal antibody infusion to the time of death or censoring at the most recent follow-up; Progression-Free Survival (PFS) was calculated from the date of the first PD-1 monoclonal antibody infusion to the time of death or first documented progression, whichever came first, or censoring at the most recent follow-up. Patients who remained alive were censored at the date of the last contact.

### Statistical analysis

Descriptive statistics were employed to summarise the genomic alterations identified in this study. The Mann–Whitney U test and Kruskal–Wallis test were used to examine differences between continuous variables, while Fisher’s exact test was utilised to compare associations between categorical variables. Survival analyses were conducted via Kaplan–Meier analysis coupled with the Log‐rank test, and the Cox’s proportional hazard model was employed to estimate the hazard ratio (HR) with a 95% confidence interval (CI). For multivariate analysis, variables with *P*-values < 0.10 in univariate analysis were included in the multivariate regression model. The consistency between genomic variations in ctDNA and tumor marker levels was assessed using the Spearman rank-order correlation coefficient. Concordance between ctDNA responses and radiological responses evaluated based on RECIST 1.1 criteria was appraised via the Kappa test. In all these tests, *P* < 0.05 was considered statistically significant. All analyses were conducted using SPSS V.22.0 or R V.3.6.3, and the results were formatted with GraphPad Prism 6.

## Results

### Characteristics of enrolled patients

Between July 2022 and June 2023, we enrolled a total of 30 patients diagnosed with advanced gastric cancer who met the specified criteria, with follow-up until January 26th, 2024. The median age of the cohort was 62 years (range, 31–75 years), consisting of 21 male patients (70%) and 9 female patients (30%). The majority of the patients (76.7%; 23/30) had tumors originating from locations other than the esophagogastric junction. Based on pathological classification, 26.7% (8/30) had moderately differentiated tumors, while 50% (15/30) had poorly differentiated tumors, including cases of signet ring cell carcinoma (16.7%; 5/30). Distant metastases were evident in 73.3% of the cohort (22/30), predominantly affecting the peritoneum (66.7%, 20/30) and liver (63.3%, 19/30). All 30 patients exhibited microsatellite stability (MSS), with one patient testing positive for EBV in situ hybridization. Initial treatment for all 30 patients involved chemotherapy in conjunction with a first-line PD-1 antibody, while 7 cases with ERBB2 amplification received additional trastuzumab. The chemotherapy regimens encompassed oxaliplatin, docetaxel, 5-fluorouracil, tegafur, and capecitabine. The PD-1 monoclonal antibodies utilised included nivolumab and sintilimab. Comprehensive clinicopathological characteristics of all patients are detailed in Table 1 and the procedure of data analysis was described in Fig. S1.

**Table 1:** Baseline clinicopathologic characteristics.

Character	Number	Percent
Number of patients	30	100.0%
Median age, years (range)	62.0 [31.0, 75.0]	
Gender		
Male	21	70.0%
Female	9	30.0%
ECOG performance status		
0	23	76.7%
1	7	23.3%
Smoking history		
Yes	17	56.7%
No	13	43.3%
Primary tumor site		
Whole stomach	3	10.0%
Gastroesophageal junction/Cardia	7	23.3%
Fundus/body	9	30.0%
Antrum	11	36.7%
Histological grade		
Moderately differentiated	8	26.7%
Poorly differentiated	15	50.0%
Unknown	7	23.3%
TNM stage		
Stage III	6	20.0%
Stage IV	24	80.0%
Metastasis		
M0	8	26.7%
M1	22	73.3%
Metastatic sites		
Liver	11	36.7%
Peritoneum	10	33.3%
Lymph nodes	9	30.0%
Bone	1	3.3%
Number of metastatic sites		
0–2	28	93.3%
≥ 3	2	6.7%
Pathological pattern		
Adenocarcinoma	25	83.3%
Signet-ring cell carcinoma	5	16.7%
HER-2 status		
Positive	7	23.3%
Negative	23	76.7%
EBV Status		
Negative	27	90.0%
Positive	1	3.3%
Unknown	2	6.7%
MSI status		
MSS	30	100.0%
TMB		
Median TMB (range)	8.10 [2.79, 30.7]	
TMB-H	11	36.7%
TMB-L	19	63.3%
Chemotherapy regimens		
SOX	21	70.0%
DX	4	13.3%
XELOX	3	10.0%
FOLFOX	2	6.7%
PD-1 monoclonal antibody		
Sintilimab	26	86.7%
Nivolumab	4	13.3%

SOX, Oxaliplatin + S-1; DX, Docetaxel + Capecitabine; XELOX, Oxaliplatin + Capecitabine; FOLFOX, Oxaliplatin + 5-Fluorouracil + Leucovorin.

In total, there were 15 responders (2 with complete responses and 13 with partial responses) and 15 non-responders (10 with stable disease and 5 with disease progression) as determined by the best overall response (BOR) according to RECIST v1.1 criteria. The Overall Response Rate (ORR), Disease Control Rate (DCR), median Progression-Free Survival (mPFS), and median Overall Survival (mOS) stood at 50%, 83.3%, 7.3 months, and not reached (NR), respectively. Stratification of the 30 patients based on the BOR assessed according to RECIST v1.1 revealed statistically significant differences in PFS (*P* < 0.001), albeit not in Overall Survival (OS), as shown in Fig. S2.

### Genomic landscape of tissue DNA and plasma ctDNA

**Figure 1:**
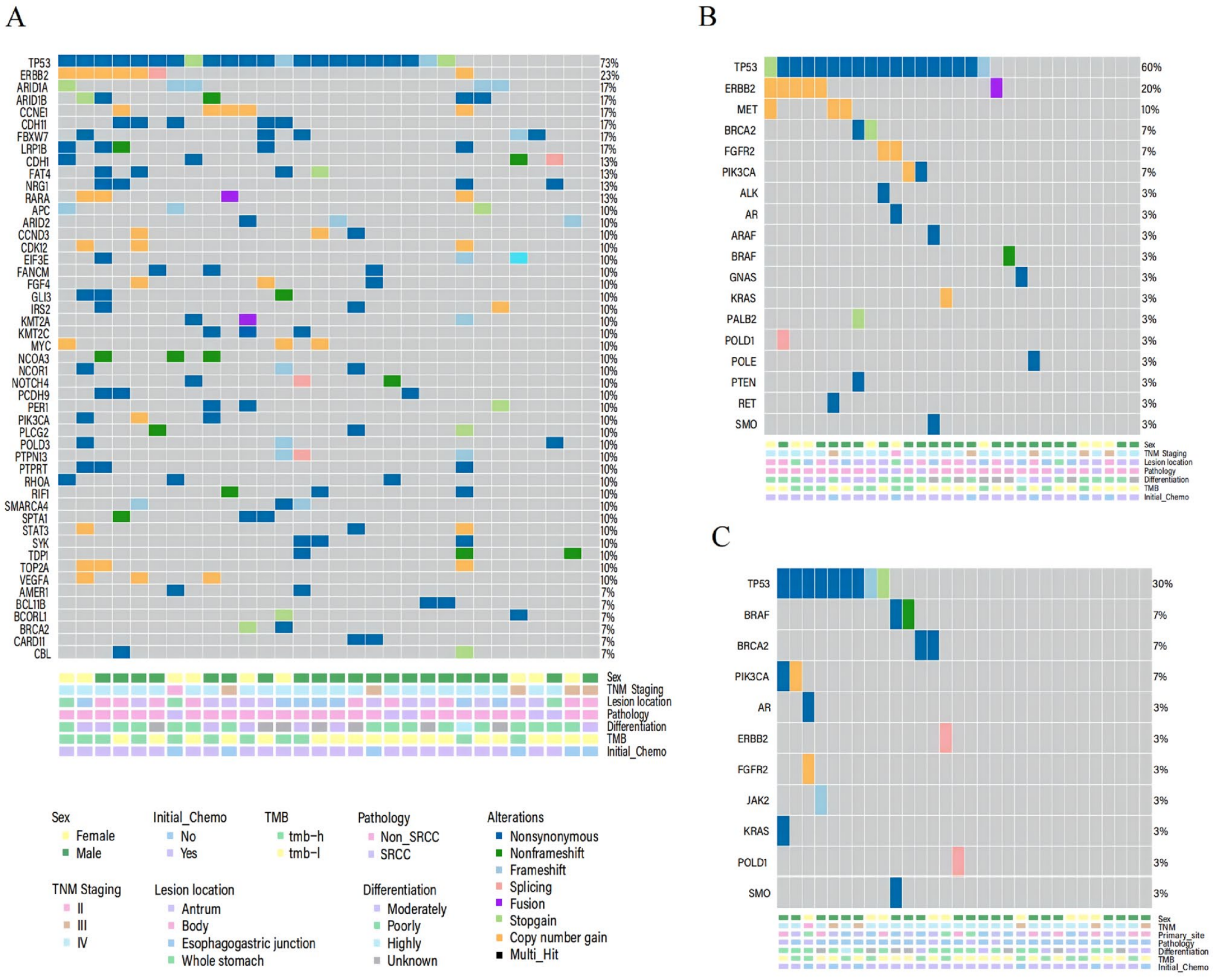
Genomic landscape of tissue DNA and plasma ctDNA: (**A**) Baseline issue; (**B**) Baseline Plasma; (**C**) Post-Treatment Plasma. TMB-H: ≥ 10Muts/Mb.

A comprehensive analysis was conducted on both plasma and tissue samples from a cohort of 30 patients. In 73.3% of the patients (22/30), genomic variations were detected in baseline plasma samples, which included specific mutations with known clinical significance as well as variants of unknown significance (VUS). Meanwhile, 26.7% of the patients (8/30) tested negative for ctDNA at baseline. Among the detected variations, TP53 mutations were the most prevalent, accounting for 60% (18/30), followed by ERBB2 (20%, 6/30), MET (10%, 3/30), BRCA2 (7%, 2/30), FGFR2 (7%, 2/30), and PIK3CA (7%, 2/30) (Fig. 1B). Additionally, in baseline tissue DNA testing of the same 30 patients, 96.7% (29/30) were found to have at least one gene mutation, with only one patient showing no gene mutations. The most frequently observed mutations were also in the TP53 gene (73.3%, 22/30), similar to the results from plasma, followed by ERBB2 (23.3%, 7/30), ARID1A (16.7%, 5/30), ARID1B (16.7%, 5/30), CCNE1 (16.7%, 5/30), and CDH11 (16.7%, 5/30) (Fig. 1A). It is worth highlighting that among the 22 patients with detectable baseline ctDNA, TP53 mutations exhibited the highest mutation frequency (refer to Supplemental Table 1). The overall concordance rate was 80.0% for TP53 (κ = 0.559) and 83.3% for ERBB2 (κ = 0.510) (Table 2).

**Table 2:** Concordance between ctDNA and tissue DNA testing (N = 30).

	ctDNA testing results	Tissue testing results		Overall concordance	*P*-value	Kappa (SE)
		Positive	Negative			
TP53	Positive	17	1	80.0%	0.001	0.559 (0.153)
	Negative	5	7			
ERBB2	Positive	4	2	83.3%	0.005	0.510 (0.190)
	Negative	3	21			

Alterations with N ≥ 6 detected in ctDNA were included in this analysis.

### Correlation between baseline TP53 VAF levels and tumor markers

**Figure 2:**
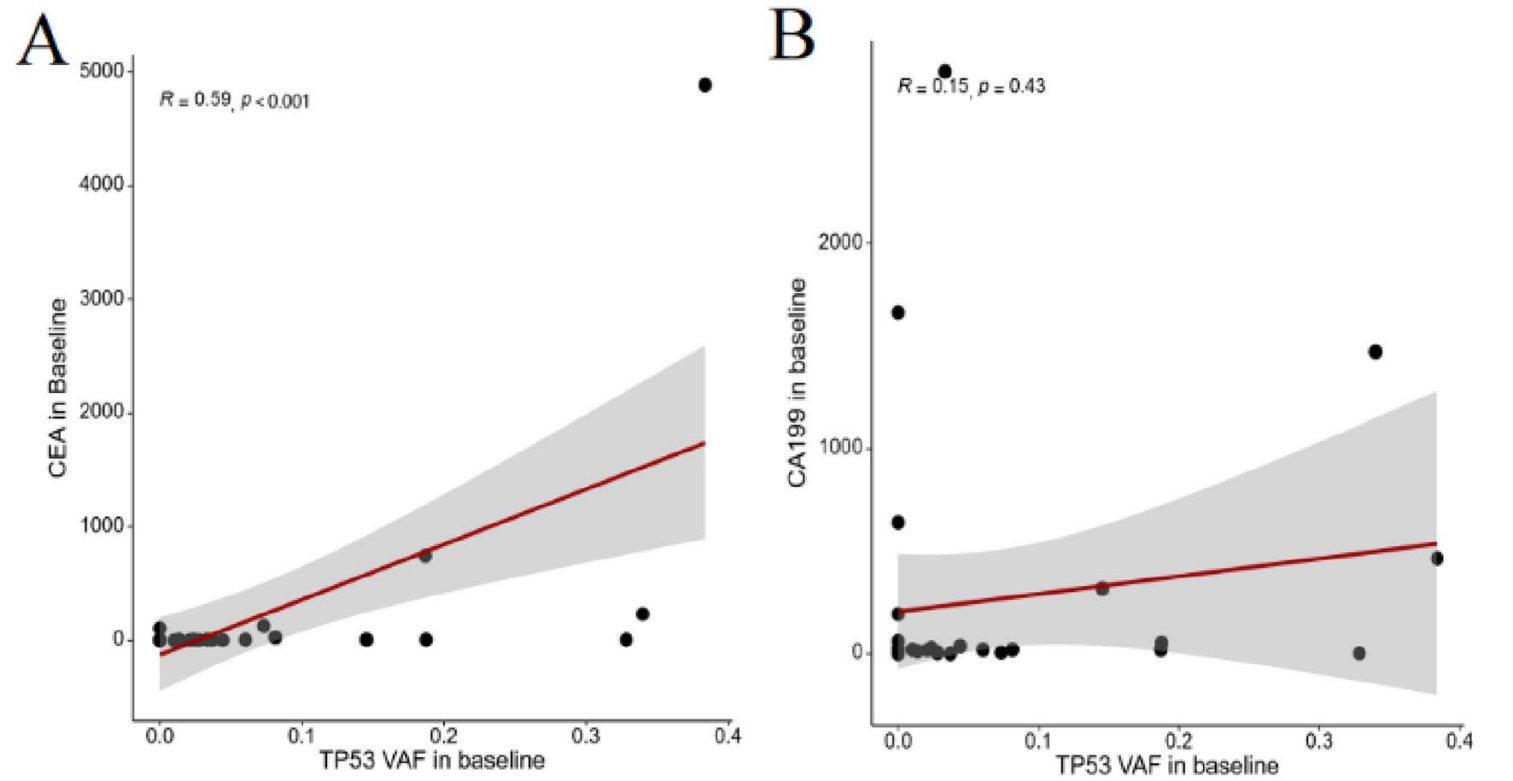
Correlation between baseline plasma TP53 VAF levels and baseline tumor marker levels assessed using Spearman’s rank correlation coefficient: (**A**) CEA; (**B**) CA19-9.

In the 22 patients where ctDNA was detected at baseline, the VAF levels of the TP53 gene were the highest (see Table S1). An exploratory analysis was conducted to examine the relationship between the VAF levels of the TP53 gene and baseline tumor markers. It was found that there is a significant positive correlation between TP53 VAF levels and the levels of CEA (Carcinoembryonic Antigen) (*P* < 0.001, Fig. 2A). However, there was no significant correlation between TP53 VAF levels and the levels of cancer antigen 19-9 (CA19-9) (Fig. 2B).

### Correlation between VAF levels pre- and post-treatment and imaging response

After two cycles of treatment, the detection rate of plasma ctDNA in the 30 patients significantly decreased from baseline, with only 50% of patients (15/30) showing genomic changes (Fig. 1C). Among the 22 patients who initially had detectable ctDNA, 9 experienced a reduction in ctDNA VAF to undetectable levels post-treatment, and all of these 9 patients achieved radiological response during treatment. Conversely, within the subgroup of 8 patients who did not have detectable ctDNA at baseline, ctDNA was detected in 2 patients after treatment; these two patients did not achieve radiological response by the end of follow-up (Table S2).

**Figure 3:**
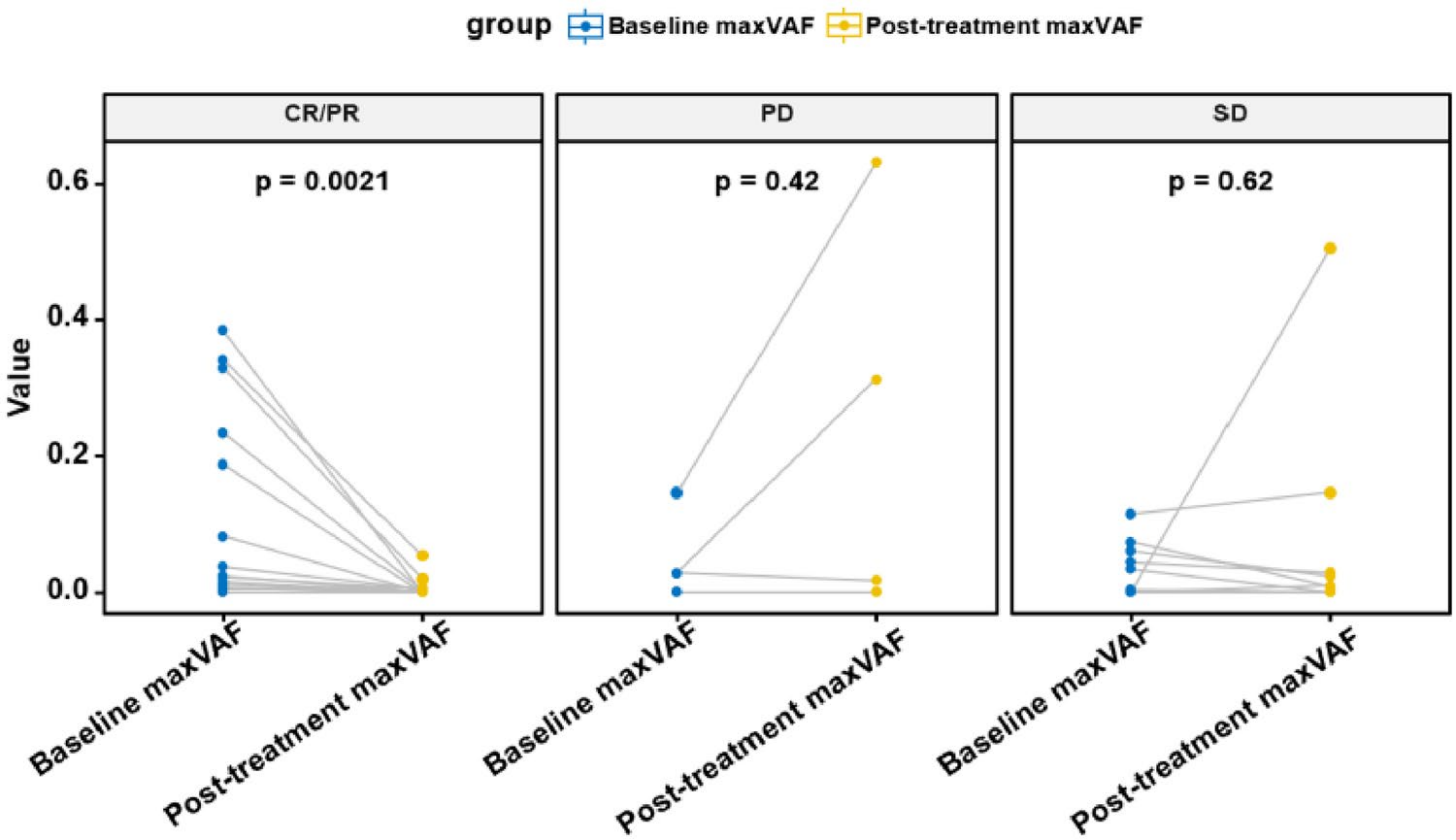
The relationship between the dynamic changes in maxVAF before and after treatment and treatment response.

Further analysis explored the relationship between baseline VAF levels, post-treatment VAF dynamics, and imaging response. It was found that baseline maxVAF levels did not statistically predict imaging response (Fig. S3). However, the dynamic changes in maxVAF post-treatment were significantly correlated with patients achieving a complete response (CR) or partial response (PR) as seen in imaging (*P* = 0.0021), while no such correlation was observed in patients with stable disease (SD) or progressive disease (PD) (Fig. 3).

### Survival analysis of advanced gastric cancer patients

The survival analysis encompassed patients diagnosed with locally advanced, metastatic, or recurrent gastric cancers (N = 30). The univariate analysis revealed no significant correlations between Progression-Free Survival (PFS) and various factors, including age, gender, TP53 gene alterations, baseline ctDNA detection, and baseline plasma maxVAF < 2.54%. Interestingly, patients with moderate differentiation, ≤ 2 metastatic sites, and a baseline plasma meanVAF < 2.29% exhibited a trend towards improved PFS, although these observations did not reach statistical significance (Hazard Ratio [HR] 0.24, 95% Confidence Interval [CI] 0.05–1.09, *P* = 0.065; HR 0.22, 95% CI 0.05–1.02, *P* = 0.052; HR 0.36, 95% CI 0.13–1.02, *P* = 0.055, respectively) (The cutoff values for maxVAF < 2.54% and meanVAF < 2.29% were based on median values). Regarding Overall Survival (OS), our analysis similarly did not identify any significant associations with the aforementioned factors. However, it is noteworthy that the presence of detectable ctDNA post-treatment showed a trend towards worse OS (HR 7.13, 95% CI 0.86–59.3, *P* = 0.069). Furthermore, lower baseline plasma maxVAF < 2.54% and meanVAF < 2.29% were linked to a tendency towards improved OS, although these findings also did not reach statistical significance (HR 0.14, 95% CI 0.02–1.19, *P* = 0.072 for both). Subsequent analysis within the multivariate model did not identify any factors with a *P*-value < 0.05. Importantly, baseline plasma meanVAF < 2.29% showed near-significant potential in predicting PFS (HR 0.08, 95% CI 0.01–1.28, *P* = 0.075), potentially serving as an independent predictor of PFS (Tables S3 and S4).

### Association of ctDNA response with radiological response and clinical outcome

**Figure 4:**
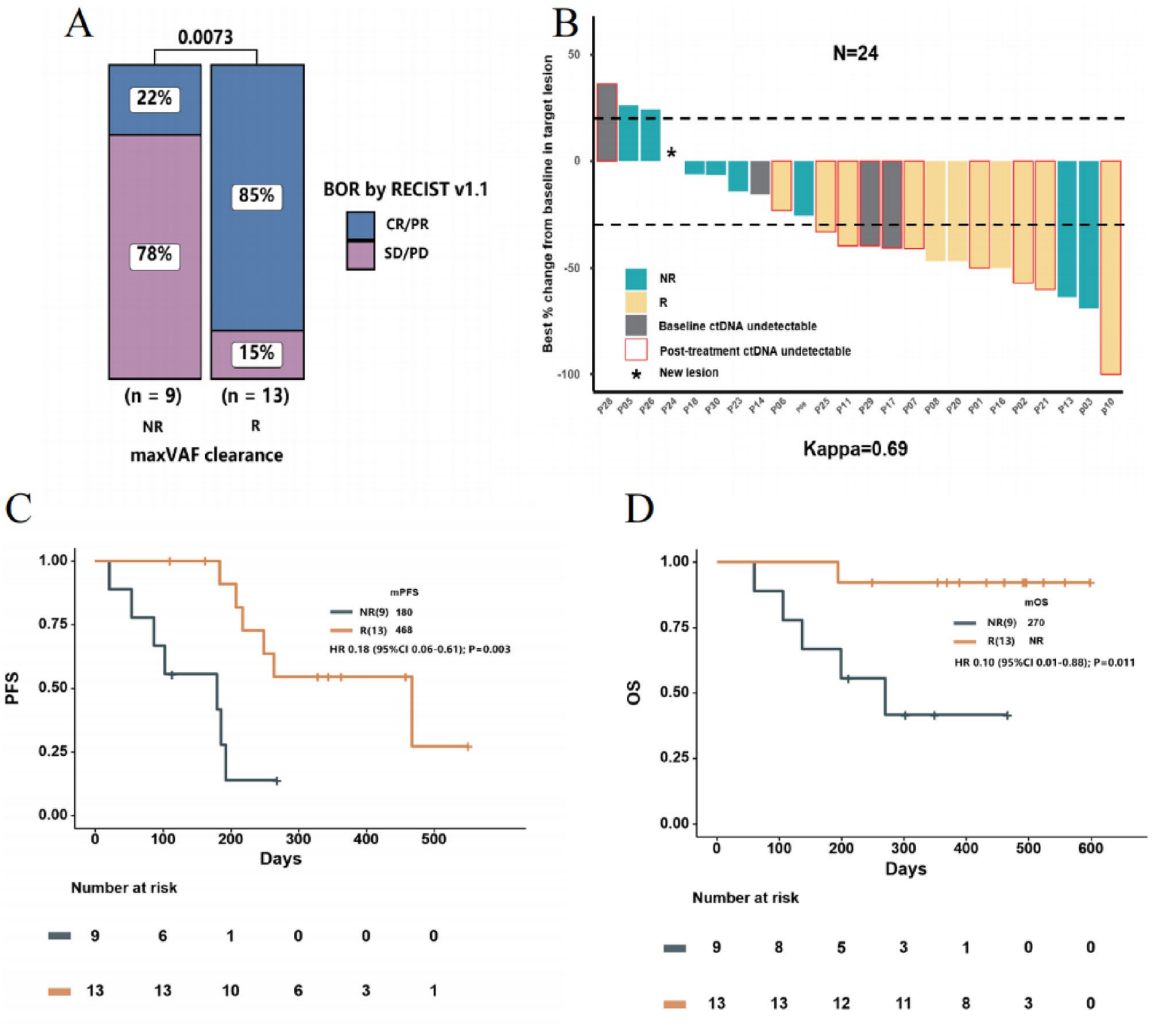
Kaplan–Meier Estimates of progression-free survival and overall survival Based on ctDNA Response. (**A**) Higher ORR observed in patients with ctDNA response, (**B**) Waterfall plot of best radiologic response and ctDNA response (only changes of measurable tumors were displayed in this figure, N = 24). (**C**), (**D**) Patients with ctDNA response had a longer PFS and OS compared to patients with non-response. ctDNA Response, maxVAF clearance. ctDNA Non-Response, maxVAF no clearance.

We assessed the correlation between early ctDNA response and radiographic response and clinical outcomes during first-line immunotherapy. Blood samples obtained from 30 patients were analysed at the first follow-up. The median time for the initial follow-up ctDNA evaluation was 7.3 weeks (interquartile range: 6.7–8.1) from the commencement of first-line therapy. Our definition of a “ctDNA response” was based on the clearance of maxVAF concerning baseline levels. It is essential to emphasise that the evaluation of ctDNA response exclusively pertained to the 22 patients with detectable ctDNA at baseline. Accordingly, these 22 patients were divided into two distinct groups: the ctDNA response group, consisting of 59.1% (13/22) of patients who achieved maxVAF clearance, and the ctDNA non-response group, encompassing the remaining 40.9% (9/22) who did not attain maxVAF clearance. Significant differences were observed between the two patient groups in terms of histological grading (*P* < 0.05); however, other baseline clinical features displayed no notable variations (Table S5). Within these two groups, as evaluated based on the Best Overall Response (BOR) according to RECIST V1.1 criteria, 85% of patients in the ctDNA response group achieved an Overall Response Rate (ORR), in contrast to only 22% in the non-response group. This disparity in ORR between the two groups reached statistical significance (*P* = 0.0073) (Fig. 4A).

Furthermore, we identified substantial agreement between ctDNA response and the best tumor shrinkage rate, as measured by Cohen’s kappa statistic (κ = 0.69), among the 24 patients with measurable lesions (Fig. 4B). Patients with a ctDNA response exhibited significantly prolonged Progression-Free Survival (PFS) in comparison to those without a ctDNA response (median PFS 15.6 months vs. 6.0 months; Hazard Ratio [HR] 0.18; 95% Confidence Interval [CI] 0.06 to 0.61; *P* = 0.003) (Fig. 4C). Similarly, Overall Survival (OS) also demonstrated a notably extended duration among ctDNA responders (median OS not reached [NR] vs. 9.0 months; HR 0.10; 95% CI 0.01 to 0.88; *P* = 0.011) (Fig. 4D). Furthermore, we subdivided the patients from the ctDNA non-response group into two subgroups: those with decreasing maxVAF and those with increasing maxVAF. Our analysis revealed that patients experiencing only a reduction in maxVAF, without achieving complete clearance, did not demonstrate a statistically significant difference in PFS and OS, compared to those with an increase in maxVAF. (refer to Fig. S4).

### Tumor tissue mutational characteristics and prognostic relevance

**Figure 5:**
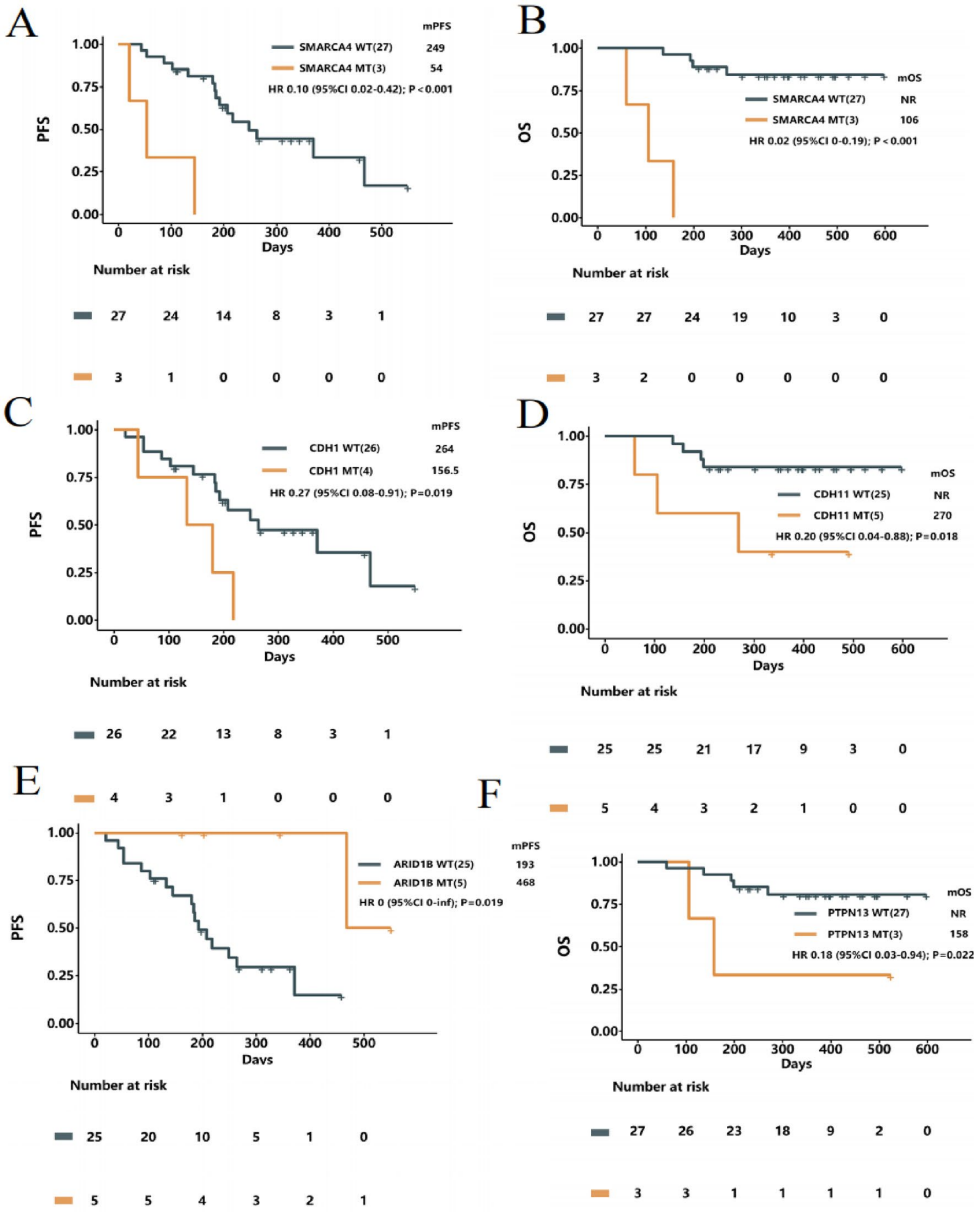
Kaplan–Meier estimates progression-free survival and overall survival according to the genomic alterations in tissue. (**A**), (**B**) Patients with SMARCA4 WT had a longer PFS and OS compared to patients with SMARCA4 MT. (**C**) Patients with CDH1 WT had a longer PFS compared to patients with CDH1 MT. (**D**) Patients with CDH11 WT had a longer OS compared to patients with CDH11 MT. (**E**) Patients with ARID1B WT had a shorter PFS compared to patients with ARID1B MT. (**F**) Patients with PTPN13 WT had a longer OS compared to patients with PTPN13 MT. WT, wild type. MT, mutation type. NR, not reached.

Upon further investigation into the correlation between baseline tumor tissue-NGS and immunotherapy prognosis, we identified a significant association between SMARCA4 mutation status and both PFS and OS. The identified mutations in SMARCA4, including p.P240Rfs63, p.G271Rfs16, and p.L925V, were associated with distinct survival outcomes. Specifically, the median PFS for patients with SMARCA4^wt^ was 8.3 months, compared to only 1.8 months for those with SMARCA4^mt^ (Hazard Ratio [HR] 0.10; 95% Confidence Interval [CI] 0.02 to 0.42; *P* < 0.001) (Fig. 5A). Additionally, the median OS was not reached (NR) in the SMARCA4^wt^ group, whereas it was 3.5 months in the SMARCA4^mt^ group (HR 0.02; 95% CI 0 to 0.19; *P* < 0.001) (Fig. 5B). Furthermore, we discovered that the mutation status of CDH1 and ARID1B influenced the PFS of immunotherapy. Mutations including CDH1 p.Y228D, p.I319_T330del, p.D514Y, c.1320 + 1G > T were identified, and the median PFS of patients with CDH1^wt^ and CDH1^mt^ were 8.8 months and 5.2 months (HR 3.7; 95% CI 1.1 to 12; *P* = 0.019) (Fig. 5C). For ARID1B, mutations such as p.S122_S123insF, p.A412G, p.R1618L, p.Q2202*, p.A412G were identified, and the median PFS of patients with ARID1B^mt^ and ARID1B^wt^ were 15.6 months and 6.4 months (*P* = 0.019; However, the HR could not be accurately calculated for the 95% CI due to the insufficient sample size and the low number of events in the ARID1B^mt^ group) (Fig. 5E). Similarly, we found that mutations in CDH11 and PTPN13 could influence Overall Survival (OS) in immunotherapy. CDH11 mutations including p.K487R, p.L384M, p.G599R, p.L479I, p.L454P were identified, and the median OS of patients with CDH11^wt^ and CDH11^mt^ were NR and 6.4 months (HR 0.2; 95% CI 0.04 to 0.88; *P* = 0.018) (Fig. 5D). For PTPN13, mutations such as p.D2209Efs6, p.M664Ifs3, c.2012 + 2T > A were identified, and the median OS of patients with PTPN13^wt^ and PTPN13^mt^ were NR and 5.3 months (HR 0.18; 95% CI 0.03 to 0.94; *P* = 0.022) (Fig. 5F).

## Discussion

In the evolving landscape of immuno-oncology, the success of immune checkpoint inhibitors (ICIs) has significantly extended the survival of patients with advanced gastric cancer^3,4,25^. Although ICIs have shown promising efficacy in a subset of these patients, a portion remains unresponsive or experiences severe and life-threatening adverse events^26^. Predicting the efficacy of ICIs more effectively could significantly enhance treatment efficiency. Consequently, identifying reliable biomarkers for ICIs is critical to refining immunotherapy approaches for gastric cancer. Traditional assessment of tumor response, such as measuring early tumor shrinkage with CT scans, although widely accepted, is expensive, involves radiation exposure, and may not accurately reflect the earliest molecular changes, particularly in tumors from cavitary organs like the stomach^27,28^. In contrast, tracking responses through circulating tumor DNA (ctDNA) offers a dynamic, non-invasive alternative that allows for the real-time monitoring of genomic alterations. This method potentially detects treatment responses or resistance sooner than conventional techniques. ctDNA testing, which can be conducted repeatedly with minimal discomfort to the patient, avoids the high costs and risks associated with frequent imaging. Despite the decreasing costs of ctDNA testing due to advancements in sequencing technologies, the clinical acceptance of imaging still predominates due to its longstanding integration into treatment protocols.

In this study, we investigated the correlation between ctDNA responses and clinical outcomes in advanced gastric cancer patients undergoing first-line therapy with combined PD-1 inhibitors and chemotherapy. While existing research indicates that ctDNA dynamics can serve as a predictive marker for immunotherapy responses, most of these studies have focused on cancers such as non-small cell lung cancer, melanoma, and Hodgkin’s lymphoma^17,29–32^. Among them, research on ctDNA in non-small cell lung cancer (NSCLC) is the most extensive. For example, Nabet and colleagues, in a study involving 46 patients with metastatic NSCLC receiving ICI monotherapy or combined with CTLA-4 inhibitors, used NGS to detect ctDNA concentrations 4 weeks after the start of treatment. They found that patients with a ≥ 50% decrease in ctDNA had longer PFS (22.4 vs. 2.3 months, HR 2.28; *P* = 0.013)^33^. In an analysis of the ATLANTIC trial, which included 66 advanced NSCLC patients treated with durvalumab, Zhang and others used NGS to measure plasma VAF at week six of treatment. They discovered that patients with a > 50% reduction in VAF had better PFS (HR 0.3, 95% CI 0.15–0.60) and OS (HR 0.29, 95% CI 0.10–0.84)^34^. Additionally, in a prospective phase II clinical trial (INSPIRE trial) that assessed ctDNA in 94 patients with various advanced solid tumors treated with pembrolizumab, patients with reduced ctDNA by NGS by the third treatment cycle showed improved ORR (42% vs. 2%), PFS (HR 0.33; 95% CI 0.19–0.58), and OS (HR 0.36; 95% CI 0.18–0.71)^35^.

Currently, research on ctDNA in the field of immunotherapy for gastric cancer is scarce, with only a few studies reported, focusing on patients after multiple lines of treatment^18,19^. For first-line treatment of advanced gastric cancer, especially the application of immune checkpoint inhibitors combined with chemotherapy, there is a lack of relevant research. In the context of first-line treatment of advanced gastric cancer with PD-1 inhibitors and chemotherapy, the exploration of ctDNA molecular response remains a field that needs continuous expansion.

In our research, we utilized a specialized next-generation sequencing (NGS) panel to analyze the Variant Allele Frequency (VAF) in patient plasma samples, aiming to identify populations exhibiting a ctDNA response. Our objectives were to establish a correlation between ctDNA response and the efficacy and long-term outcomes of first-line PD-1 inhibitor therapy combined with chemotherapy. Previous studies have employed various metrics, such as meanVAF, maxVAF, or medianVAF, to monitor ctDNA dynamics, and there is currently no clear consensus on the standards for defining ctDNA response, which may include reductions in ctDNA levels, ctDNA clearance, or the ratio of VAF during treatment to baseline VAF^34,36,37^. Given the limited scope of our ctDNA-NGS study, relying solely on meanVAF changes as an indicator of ctDNA response could result in less reliable outcomes. We hypothesized that maxVAF typically reflects the most significant tumor-specific variations in the samples, thereby providing a more accurate indication of dominant mutation burden. In contrast, meanVAF might be diluted by lower-frequency mutations, potentially rendering it less sensitive than maxVAF. Furthermore, a comparative summary analysis of ctDNA dynamics in non-small cell lung cancer immunotherapy suggested that changes in maxVAF are the best predictors of treatment response^30^. Literature also indicates that patients with ctDNA clearance (rather than reduction) generally have a better prognosis^35,38^. Therefore, in our study, ctDNA response was defined as the clearance of maxVAF. Ultimately, we confirmed that maxVAF clearance is closely associated with optimal treatment response in immunotherapy, with patients demonstrating maxVAF clearance experiencing longer PFS and OS.

This study, as a prospective research, collected plasma samples from patients before the start of treatment, allowing for a more accurate tracking of the temporal sequence between changes in ctDNA levels and treatment responses. This ensures the integrity and consistency of the data, helping to reduce the biases and limitations often encountered in retrospective studies. However, this study still has certain limitations. Firstly, the small sample size included may introduce a degree of bias. Secondly, there are limitations associated with the ctDNA detection methods themselves. The amount of ctDNA released can be affected by various factors, including the type, size, location of the tumor, and its microenvironment. In early-stage tumors or those with low tumor burden, ctDNA abundance may be extremely low, posing a significant challenge to the sensitivity of detection technologies^39^. Moreover, with age, clonal expansion of normal hematopoietic cells may carry cancer-related genetic variations, known as clonal hematopoiesis, which could decrease the specificity of ctDNA detection since non-tumor-derived variations could be confused with ctDNA variations^40^. Despite the high sensitivity and specificity of ctDNA detection technologies such as dPCR and NGS, technical noise and errors (e.g., sample processing, DNA extraction, PCR amplification) can still affect the accuracy of results, leading to false positives or negatives^41^. Therefore, future efforts are needed to develop more advanced ctDNA detection technologies and methods, establish more comprehensive testing and analysis standards, and validate the value of ctDNA applications through large-scale clinical trials.

In our study, we also employed a comprehensive 737-gene panel for tumor tissue-based next-generation sequencing, aiming to explore the relationship between primary tumor genomic alterations and the long-term efficacy of immunotherapy in advanced gastric cancer. We discovered that patients with SMARCA4 mutations exhibited significantly reduced PFS and OS following immunotherapy. This observation is consistent with similar studies conducted on advanced non-small cell lung cancer, which also reported reduced survival rates associated with SMARCA4 mutations^42^. These findings underscore the potential negative prognostic impact of SMARCA4 mutations on survival outcomes in cancer patients treated with immunotherapy, highlighting a critical area for further research to understand the underlying mechanisms.

Conversely, our analysis identified that patients with ARID1B mutations experienced a significant extension in PFS following immunotherapy, this may be because ARID1B, as part of the SWI/SNF chromatin remodeling complex, leads to increased genomic instability due to mutations. Tumors with chromatin remodeling defects sometimes have a higher mutation burden, which is often associated with a better response to immunotherapy^43^. Additionally, a large-scale data analysis of lung adenocarcinoma confirmed that ARID1B mutations are a major reason for the sensitive response of lung adenocarcinoma patients to immunotherapy^44^. However, the impact of ARID1B mutations on immunotherapy for gastric cancer still needs further exploration.

This suggests that ARID1B mutations may confer a survival advantage under immunotherapeutic treatment, pointing to the potential for these mutations as biomarkers for favorable responses to immunotherapy. Additionally, we noted that patients with CDH11 mutations had prolonged OS when treated with immunotherapy. Given the established role of CDH11 in enhancing cellular proliferation and metastasis in various cancers^45–47^, this outcome prompts intriguing questions about the interplay between CDH11 mutations and immune response modulation. The apparent contradiction between the known effects of CDH11 on cancer progression and its association with improved survival in the context of immunotherapy warrants further exploration to clarify its role and mechanism in enhancing immunotherapy efficacy. Although our results have found mutations associated with the prognosis of patients with advanced gastric cancer receiving immunotherapy, the limited sample size and the relatively small number of occurrences for each specific mutation in our study may lead to overestimations or underestimations of their true effects. Therefore, we advocate for future research involving larger cohorts, which will allow us to more accurately assess the prognostic value of specific genomic alterations in similar settings, and thereby enable more reliable conclusions about the role of these genetic mutations in the context of immunotherapy.

Our dual sequencing approach, integrating both tissue and plasma sample NGS, aims to leverage the strengths and address the limitations of each method. NGS of tissue samples faces challenges of temporal and spatial heterogeneity: since a single tissue sample NGS cannot reflect the evolution of the tumor genome over time and the genetic heterogeneity between different areas of the same tumor or between primary and metastatic sites, this limits its ability to capture the full diversity of tumor genetics. In contrast, plasma ctDNA testing can provide a dynamic view of tumor genome mutations, thereby complementing the limitations of tissue NGS. Plasma ctDNA reflects real-time changes in the tumor genome as it progresses and responds to treatment, providing us with a more comprehensive view of the tumor’s genetic landscape. Thus, although tissue NGS provides a detailed picture of tumor genetic heterogeneity, the analysis of plasma ctDNA offers a valuable complement to understanding the dynamic genetic changes in tumors. This integrated approach underscores the importance of combining data from different samples to gain a more comprehensive understanding of tumor behavior and guide personalized treatment strategies.

## Conclusion

Our study applied NGS to track early quantitative ctDNA changes in the plasma of advanced gastric cancer patients receiving first-line immunotherapy and chemotherapy, successfully demonstrated that ctDNA response is a potential biomarker for predicting the efficacy and prognosis of first-line PD-1 inhibitor therapy combined with chemotherapy.

### Supplementary Information

Supplementary Figure S1.

Supplementary Figure S2.

Supplementary Figure S3.

Supplementary Figure S4.

Supplementary Information.

## Data Availability

All the data that support the findings of this study are available from the corresponding authors upon reasonable request.

## Abbreviations

BOR: Best overall response
CA19-9: Cancer antigen 19-9
CEA: Carcinoembryonic antigen
CI: Confidence interval
CR: Complete response
ctDNA: Circulating tumor DNA
CTLA4: Cytotoxic T lymphocyte associated protein 4
DCR: Disease control rate
dMMR: Mismatch repair deficiency
EBV: Epstein–Barr virus
ECOG: Eastern cooperative oncology group
FFPE: Formalin-fixed paraffin-embedded
H&E: Hematoxylin and eosin
HER-2: Human epidermal growth factor receptor 2
HR: Hazard ratio
ICI: Immune checkpoint inhibitor
Indels: Insertions and deletions
MSI/MSS: Microsatellite instability/microsatellite stability
MT: Mutation type
NGS: Next-generation sequencing
NR: Not reached
ORR: Objective response rate
OS/mOS: Overall survival/median overall survival
PD-1: Programmed cell death-receptor 1
PD-L1: Programmed cell death-ligand 1
PFS/mPFS: Progression-free survival/median progression-free survival
PD: Progressive disease
PR: Partial response
PS: Performance status
SD: Stable disease
SNVs: Single nucleotide variations
TMB/TMB-H/TMB-L: Tumor mutational burden/high-level tumor mutational burden/low-level tumor mutational burden
VAF/maxVAF: Variant allele frequencies/maximum variant allele frequencies
VUS: Variants of unknown significance
WT: Wild type
