# Supplementary figures and images for: Plasma circulating tumor DNA unveils the efficacy of PD-1 inhibitors and chemotherapy in advanced gastric cancer
Source: Sci Rep. 2024 Jun 18;14:14027. doi: 10.1038/s41598-024-63486-x (PMC11189402; doi:10.1038/s41598-024-63486-x)

# Figure S1

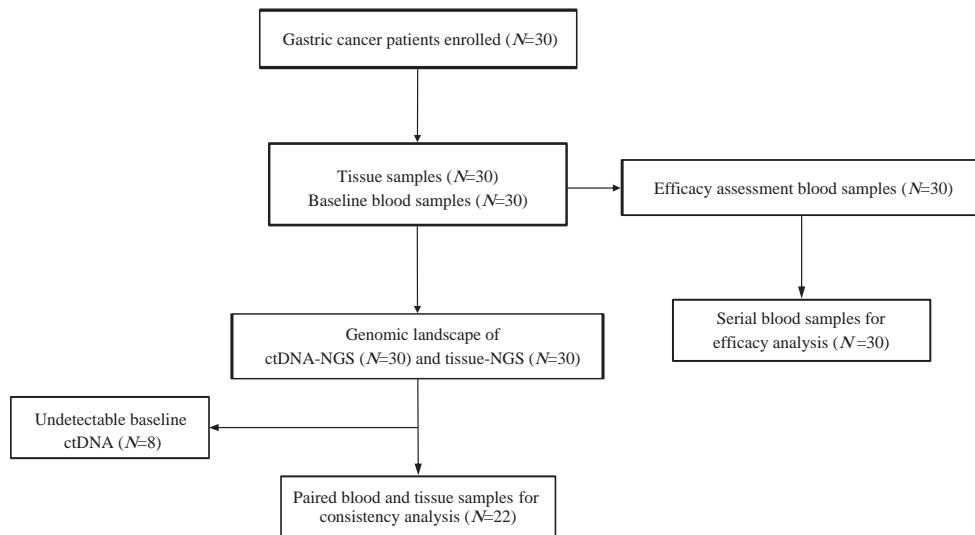

Supplement: Supplementary file 1 — Supplementary Figure S1. [file 41598_2024_63486_MOESM1_ESM.pdf]

Figure S2

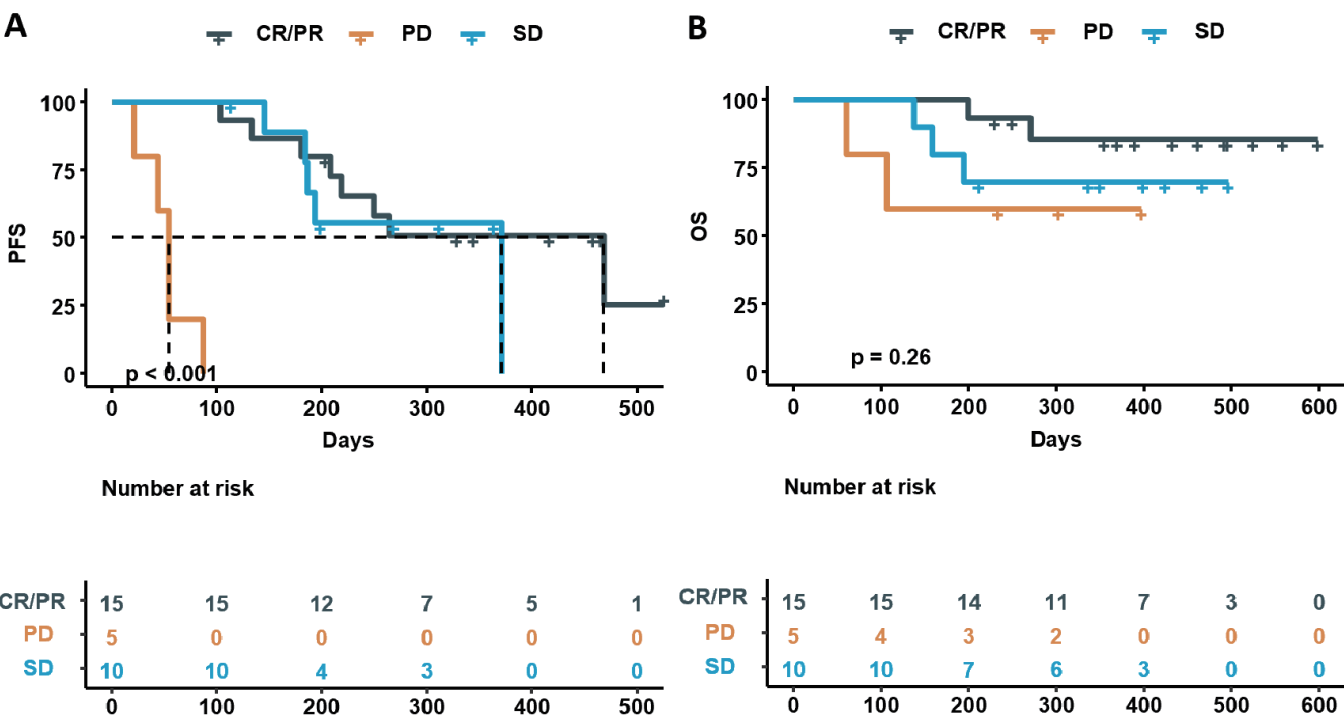

Supplement: Supplementary file 2 — Supplementary Figure S2. [file 41598_2024_63486_MOESM2_ESM.pdf]

Figure S3

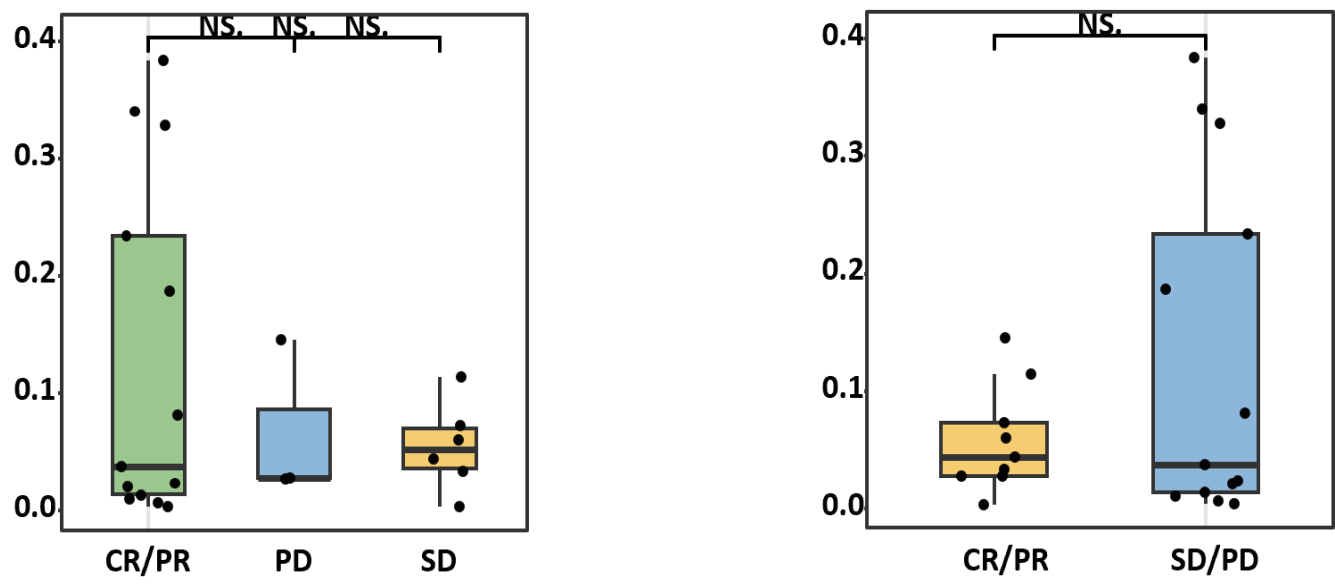

Supplement: Supplementary file 3 — Supplementary Figure S3. [file 41598_2024_63486_MOESM3_ESM.pdf]

Figure S4

A

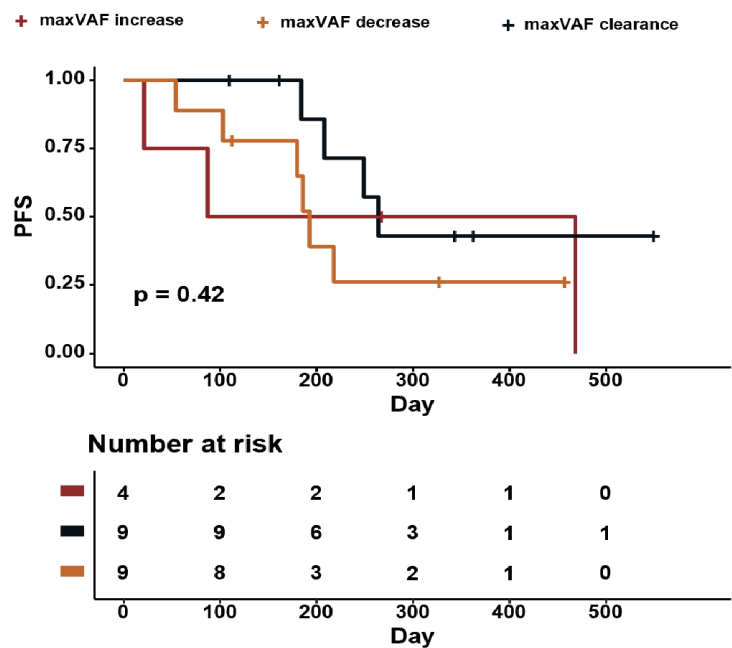

B

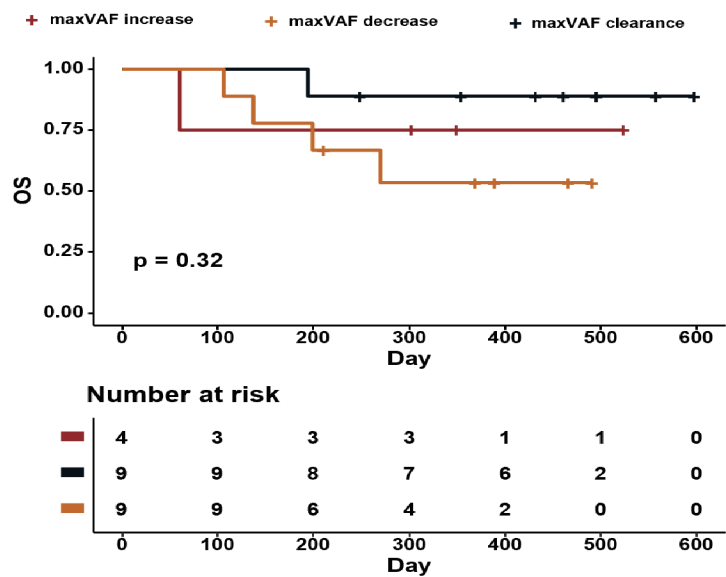

Supplement: Supplementary file 4 — Supplementary Figure S4. [file 41598_2024_63486_MOESM4_ESM.pdf]
