# Supplementary material for: Plasma circulating tumor DNA unveils the efficacy of PD-1 inhibitors and chemotherapy in advanced gastric cancer
Source: Sci Rep. 2024 Jun 18;14:14027. doi: 10.1038/s41598-024-63486-x (PMC11189402; doi:10.1038/s41598-024-63486-x)
Supplement: Supplementary file 5 — Supplementary Information. [file 41598_2024_63486_MOESM5_ESM.docx]

**Supplemental Information**

**Article title**

Plasma Circulating Tumor DNA Unveils the Efficacy of PD-1 Inhibitors and Chemotherapy in Advanced Gastric Cancer

**Author names and affiliation**

**Rongqi Jiang^1,2,3^, Xu Cheng^1^, Ping Li^1^, Enqing Meng^1^, Xinyi Wu^1^, Hao Wu^*1,2,3^**

^1^Department of Oncology, The First Affiliated Hospital of Nanjing Medical University, Nanjing, China.

^2^Gastric Cancer Center, The First Affiliated Hospital of Nanjing Medical University, Nanjing, China.

^3^Institute for Gastric Cancer Research, Nanjing Medical University, Nanjing, China.

^*^Correspondence: Hao Wu, Gastric Cancer Center, The First Affiliated Hospital of Nanjing Medical University, 300 Guangzhou Road, Nanjing 210029, Jiangsu Province, PR China. Email: whdactor@njmu.edu.cn

**Supplementary Table 1. Concordance between** **baseline plasma NGS and tissue NGS analysis**

| Patient ID | Mutation identified from ctDNA | baseline plasma variant allele frequencies | Tissue NGS | Consistent Detection of Maximum variant Allele Frequency for Somatic Variants |
| --- | --- | --- | --- | --- |
| 1 | TP53_c.713G>A BRCA2_c.289G>T | 0.383663 0.153574 | Identified Identified | yes |
| 2 | TP53_c.818G>A PALB2_c.2257C>T BRCA2_c.8787G>C PTEN_c.830C>A | 0.0103426 0.00610376 0.0045045 0.00334793 | Identified Identified Not identified Not identified | yes |
| 3 | TP53_c.708C>G | 0.32819 | Identified | yes |
| 4 | GNAS_c.608T>C | 0.00381679 | Not identified | no |
| 5 | TP53_c.467G>C | 0.145253 | Identified | yes |
| 6 | POLE_c.4411C>G | 0.00336384 | Not identified | no |
| 7 | TP53_c.711G>A | 0.0813107 | Identified | yes |
| 8 | TP53_c.314G>T POLD1_c.1776-2A>C | 0.0372777 0.00740933 | Identified Not identified | yes |
| 9 | TP53_c.821T>G PIK3CA_c.1633G>A | 0.0439883 0.0382912 | Identified Not identified | yes |
| 10 | TP53_c.797G>A | 0.0235092 | Identified | yes |
| 11 | TP53_c.796G>A ALK_c.2194G>A | 0.0137073 0.00460553 | Identified Not identified | yes |
| 13 | AR_c.2169G>T TP53_c.818G>A | 0.233781 0.187332 | Identified Identified | yes |
| 16 | TP53_c.455C>T ERBB2_fusion | 0.339966 0.0857293 | Identified Identified | yes |
| 18 | TP53_c.527G>A RET_c.889C>A | 0.0730669 0.0301308 | Identified Not identified | yes |
| 20 | TP53_c.485T>A ARAF_c.671C>T SMO_c.1418A>G | 0.0207023 0.0148348 0.00407595 | Identified Not identified Not identified | yes |
| 21 | TP53_c.451C>T | 0.186889 | Identified | yes |
| 23 | BRAF_c.95_100dup | 0.114175 | Not identified | no |
| 24 | TP53_c.742C>T | 0.0277848 | Identified | yes |
| 25 | ERBB2 fusion | 0.00663193 | Not identified | no |
| 26 | TP53_c.164_180del | 0.027323 | Identified | yes |
| 27 | TP53_c.818G>A | 0.0332843 | Not identified | no |
| 30 | TP53_c.818G>A | 0.0601255 | Identified | yes |

**Supplementary Table 2. Treatment strategies, ctDNA testing results, and clinical outcomes in 30 patients with gastric cancer**

| **ID** | **Age** | **Gender** | **First-line regimens** | **Baseline  ctDNA detectable** | **Post-treatment  ctDNA detectable** | **ctDNA response** | **OR*** | **PFS status** | **PFS months** |
| --- | --- | --- | --- | --- | --- | --- | --- | --- | --- |
| P1 | 59 | Female | Sintilimab+SOX | YES | NO | R | PR | 1 | 6.9 |
| P2 | 59 | male | Sintilimab+SOX | YES | NO | R | PR | 0 | 18.3 |
| P3 | 70 | Female | Sintilimab+Trastuzumab+SOX | YES | YES | NR | PR | 1 | 6.0 |
| P4 | 67 | male | Sintilimab+Trastuzumab+SOX | YES | YES | R | CR | 1 | 15.6 |
| P5 | 65 | male | Sintilimab+XELOX | YES | YES | NR | PD | 1 | 0.7 |
| P6 | 74 | male | Sintilimab+XELOX | YES | NO | R | SD | 0 | 12.1 |
| P7 | 69 | male | Nivolumab+Trastuzumab+SOX | YES | NO | R | PR | 0 | 5.4 |
| P8 | 67 | male | Sintilimab+Trastuzumab+SOX | YES | YES | R | PR | 0 | 15.3 |
| P9 | 40 | male | Sintilimab+SOX | YES | YES | NR | SD | 1 | 6.2 |
| P10 | 59 | male | Sintilimab+SOX | YES | NO | R | CR | 0 | 3.7 |
| P11 | 52 | male | Sintilimab+SOX | YES | NO | R | PR | 1 | 8.8 |
| P12 | 35 | Female | Sintilimab+SOX | NO | YES | NA | SD | 1 | 12.4 |
| P13 | 65 | Female | Nivolumab+SOX | YES | YES | NR | PR | 1 | 3.4 |
| P14 | 33 | Female | Sintilimab+DX | NO | NO | NA | SD | 0 | 6.6 |
| P15 | 31 | Female | Sintilimab+DX | NO | NO | NA | PD | 1 | 1.5 |
| P16 | 63 | Female | Sintilimab+Trastuzumab+FOLFOX | YES | YES | R | PR | 1 | 7.3 |
| P17 | 72 | male | Sintilimab+FOLFOX | NO | NO | NA | PR | 1 | 4.4 |
| P18 | 59 | male | Sintilimab+DX | YES | YES | NR | SD | 0 | 3.8 |
| P19 | 75 | male | Sintilimab+SOX | NO | YES | NA | SD | 1 | 4.8 |
| P20 | 75 | male | Sintilimab+SOX | YES | YES | R | PR | 0 | 10.9 |
| P21 | 61 | Female | Nivolumab+Trastuzumab+XELOX | YES | NO | R | PR | 0 | 11.5 |
| P22 | 65 | male | Sintilimab+SOX | NO | NO | NA | SD | 0 | 10.4 |
| P23 | 59 | male | Sintilimab+SOX | YES | YES | NR | SD | 0 | 8.9 |
| P24 | 46 | male | Sintilimab+SOX | YES | YES | NR | PD | 1 | 2.9 |
| P25 | 61 | male | Sintilimab+Trastuzumab+SOX | YES | NO | R | PR | 1 | 8.3 |
| P26 | 66 | Female | Sintilimab+SOX | YES | YES | NR | PD | 1 | 1.8 |
| P27 | 57 | male | Nivolumab+SOX | YES | NO | R | SD | 1 | 6.1 |
| P28 | 75 | male | Sintilimab+SOX | NO | NO | NA | PD | 1 | 1.8 |
| P29 | 58 | male | Sintilimab+SOX | NO | NO | NA | PR | 0 | 6.8 |
| P30 | 64 | male | Sintilimab+DX | YES | YES | NR | SD | 1 | 6.4 |

CR, complete response; DX, Docetaxel+Capecitabine; FOLFOX, Oxaliplatin+5-Fluorouracil+Leucovorin; NA, not applicable; NR: ctDNA non-response; PR, partial response; PD, progressive disease; R: ctDNA response; SD, stable disease; SOX, Oxaliplatin+S-1; XELOX, Oxaliplatin+Capecitabine.

*OR, best objective response assessed during treatment.

For PFS status, 0=progression free; 1=progressed.

**Supplementary Table 3. Univariate and multivariate analyses of progression-free survival relative to clinical variables and plasma ctDNA detection outcomes**

|  | PFS univariate analysis | | PFS multivariate analysis** | |
| --- | --- | --- | --- | --- |
| Characteristics | HR (95%CI) | P-value | HR (95%CI) | P-value |
| Age |  |  |  |  |
| ≥62(n=15) vs＜62(n=15) | 1.41(0.56-3.59) | 0.47 | - | - |
| Gender |  |  |  |  |
| Women(n=9) vs men(n=21) | 1.86(0.70-4.90) | 0.205 | - | - |
| Differentiation |  |  |  |  |
| Moderately(n=8) vs Poorly(n=15) | 0.24(0.05-1.09) | 0.065 | 0.13(0.01-1.68) | 0.119 |
| Number of metastatic sites |  |  |  |  |
| 0-2(n=28) vs ≥3(n=2) | 0.22(0.05-1.02) | 0.052 | 0.66(0.10-4.45) | 0.665 |
| TP53 gene alteration |  |  |  |  |
| Yes(n=18) vs Not(n=12) | 1.29(0.50-3.34) | 0.596 | - | - |
| Baseline ctDNA detectable |  |  |  |  |
| Yes(n=22) vs Not(n=8) | 0.71(0.25-2.02) | 0.521 | - | - |
| Post-treatment ctDNA detectable |  |  |  |  |
| Yes(n=15) vs Not(n=15) | 1.62(0.63-4.22) | 0.314 | - | - |
| Baseline plasma maxVAF＜2.54%* |  |  |  |  |
| Yes(n=15) vs Not(n=15) | 0.50(0.19-1.32) | 0.153 | - | - |
| Baseline plasma meanVAF＜2.29%* |  |  |  |  |
| Yes(n=15) vs Not(n=15) | 0.36(0.13-1.02) | 0.055 | 0.08(0.01-1.28) | 0.075 |
| Post-treatment maxVAF clearance |  |  |  |  |
| Yes(n=13) vs Not(n=9) | 0.18(0.06-0.61) | 0.006 | 0.27(0.03-2.87) | 0.278 |

*Used the median value as a cut-off.

**Variables with P-values < 0.10 in univariate analysis (log-rank test) were included in multivariate analysis.

**Supplementary Table 4. Univariate analyses of overall survival relative to clinical variables and plasma ctDNA detection outcomes**

|  | OS univariate analysis | |
| --- | --- | --- |
| Characteristics | HR (95%CI) | P-value |
| Age |  |  |
| ≥62(n=15) vs＜62(n=15) | 2.77(0.54-14.3) | 0.203 |
| Gender |  |  |
| Women(n=9) vs men(n=21) | 1.67(0.37, 7.48) | 0.497 |
| Differentiation |  |  |
| Moderately(n=8) vs Poorly(n=15) | 0.33(0.04-2.85) | 0.291 |
| Number of metastatic sites |  |  |
| 0-2(n=28) vs ≥3(n=2) | 0.27(0.03-2.25) | 0.194 |
| TP53 gene alteration |  |  |
| Yes(n=18) vs Not(n=12) | 4.38(0.53, 36.4) | 0.135 |
| Baseline ctDNA detectable |  |  |
| Yes(n=22) vs Not(n=8) | 2.28(0.27, 18.9) | 0.434 |
| Post-treatment ctDNA detectable |  |  |
| Yes(n=15) vs Not(n=15) | 7.13(0.86, 59.3) | 0.069 |
| Baseline plasma maxVAF＜2.54%* |  |  |
| Yes(n=15) vs Not(n=15) | 0.14(0.02, 1.19) | 0.072 |
| Baseline plasma meanVAF＜2.29%* |  |  |
| Yes(n=15) vs Not(n=15) | 0.14(0.02, 1.19) | 0.072 |
| Post-treatment maxVAF clearance |  |  |
| Yes(n=13) vs Not(n=9) | 0.10(0.01, 0.88) | 0.038 |

*Used the median value as a cut-off.

**Supplementary Table 5. Clinical characteristics between patients of NR and R groups at first-line treatment**

| Characteristic | NR | R | P-value |
| --- | --- | --- | --- |
|  | N = 9 | N = 13 |  |
| Age | 64 (40, 70) | 61 (52, 75) | 0.6 |
| Gender |  |  | 0.7 |
| Male | 6 (67%) | 10 (77%) |  |
| Female | 3 (33%) | 3 (23%) |  |
| Primary tumor site |  |  | 0.7 |
| Whole stomach | 1 (11%) | 1 (7.7%) |  |
| Gastroesophageal junction/cardia | 1 (11%) | 4 (31%) |  |
| Fundus/body | 3 (33%) | 5 (38%) |  |
| Antrum | 4 (44%) | 3 (23%) |  |
| Histological grade |  |  | 0.045 |
| Moderately differentiated | 0 (0%) | 7 (54%) |  |
| Poorly differentiated | 6 (67%) | 4 (31%) |  |
| Unknown | 3 (33%) | 2 (15%) |  |
| TNM stage |  |  | 0.4 |
| Stage III | 3 (33%) | 1 (7.7%) |  |
| Stage IV | 6 (67%) | 12 (92%) |  |
| Metastasis |  |  | 0.6 |
| M0 | 3 (33%) | 2 (15%) |  |
| M1 | 6 (67%) | 11 (85%) |  |
| Chemotherapy regimens |  |  | 0.3 |
| DX | 2 (22%) | 0 (0%) |  |
| FOLFOX | 0 (0%) | 1 (7.7%) |  |
| SOX | 6 (67%) | 10 (77%) |  |
| XELOX | 1 (11%) | 2 (15%) |  |
| PD-1 inhibitors |  |  | 0.8 |
| Nivolumab | 1 (11%) | 3 (23%) |  |
| Sintilimab | 8 (89%) | 10 (77%) |  |

NR: ctDNA non-response; R: ctDNA response.

**Supplementary Table 6. 47-gene NGS panel that used for plasma circulating tumor DNA sequencing**

| AKT1 | ALK | AR | ARAF | BRAF |
| --- | --- | --- | --- | --- |
| BRCA1 | BRCA2 | CDK4 | CDKN2A | EGFR |
| ERBB2 | ERBB3 | FGFR1 | FGFR2 | FGFR3 |
| GNA11 | GNAQ | HRAS | IDH1 | IDH2 |
| JAK2 | JAK3 | KIT | KRAS | MAP2K1 |
| MAP2K2 | MET | MTOR | NRAS | NRG1 |
| NTRK1 | NTRK2 | NTRK3 | PALB2 | PDGFRA |
| PDGFRB | PIK3CA | POLD1 | POLE | PTEN |
| RAF1 | RET | ROS1 | SMO | STK11 |
| TP53 | GNAS |  |  |  |

**Supplementary Table 7. 737-gene NGS panel that used for tissue tumor DNA sequencing.**

| ABL1 | ABRAXAS1 | AKT1 | AKT2 | AKT3 | ALK | APC |
| --- | --- | --- | --- | --- | --- | --- |
| AR | ARAF | ARID1A | ATM | ATR | AURKA | BARD1 |
| BCL2L11 | BRAF | BRCA1 | BRCA2 | BRIP1 | BTK | CCND1 |
| CCND3 | CD274 | CDK12 | CDK4 | CDK6 | CDKN1B | CDKN2A |
| CDKN2B | CHD1 | CHEK1 | CHEK2 | CRBN | CSF1R | CTNNB1 |
| DDR2 | DNMT3A | EGFR | EPCAM | EPHA2 | EPHA3 | ERBB2 |
| ERBB3 | ERBB4 | ERCC3 | ERRFI1 | ESR1 | EZH2 | FANCE |
| FANCL | FAT1 | FBXW7 | FGF3 | FGF4 | FGFR1 | FGFR2 |
| FGFR3 | FGFR4 | FLCN | FLT1 | FLT3 | FLT4 | FOXA1 |
| FRS2 | GEN1 | GLI1 | GLI2 | GLI3 | GNA11 | GNAQ |
| GNAS | HDAC2 | HGF | HOXB13 | HRAS | IDH1 | IDH2 |
| IGF1R | IGF2 | IL7R | INPP4B | JAK1 | JAK2 | JAK3 |
| KDR | KEAP1 | KIT | KRAS | LRP1B | MAP2K1 | MAP2K2 |
| MCL1 | MDM2 | MDM4 | MET | MLH1 | MLH3 | MRE11 |
| MSH2 | MSH6 | MTOR | MYC | MYCN | NBN | NF1 |
| NF2 | NFKBIA | NKX2-1 | NRAS | NRG1 | NTRK1 | NTRK2 |
| NTRK3 | PALB2 | PBRM1 | PCDH9 | PDCD1LG2 | PDGFRA | PDGFRB |
| PIK3CA | PIK3R1 | PIK3R2 | PLCG2 | PLXNA1 | PML | PMS2 |
| POLD1 | POLE | PPP2R2A | PTCH1 | PTEN | RAC1 | RAD50 |
| RAD51 | RAD51B | RAD51D | RAD54L | RAF1 | RARA | RB1 |
| RET | RICTOR | RNF43 | ROS1 | RPTOR | RXRA | SETD2 |
| SMARCA4 | SMARCB1 | SMO | SRC | STAG2 | STK11 | SYK |
| TERT | TP53 | TSC1 | TSC2 | VEGFA | VHL | ZBTB16 |
| ZNRF3 | AEN | ALKBH2 | ALKBH3 | APEX1 | APEX2 | APLF |
| APTX | ATRIP | ATRX | BLM | CCNH | CDK7 | CENPS |
| CETN2 | CHAF1A | CLK2 | CUL3 | CUL4A | CUL5 | DCLRE1A |
| DCLRE1B | DCLRE1C | DDB1 | DDB2 | DMC1 | DNTT | DUT |
| EME1 | EME2 | ENDOV | ERCC1 | ERCC2 | ERCC4 | ERCC5 |
| ERCC6 | ERCC8 | EXO1 | FAAP100 | FAAP20 | FAAP24 | FAN1 |
| FANCA | FANCB | FANCC | FANCD2 | FANCF | FANCG | FANCI |
| FANCM | FEN1 | GTF2H1 | GTF2H3 | GTF2H4 | GTF2H5 | H2AX |
| HELQ | HES1 | HFM1 | HLTF | HMGB1 | HUS1 | LIG1 |
| LIG3 | LIG4 | MAD2L2 | MBD4 | MDC1 | MGMT | MMS19 |
| MNAT1 | MPG | MPLKIP | MSH3 | MSH4 | MSH5 | MUS81 |
| MUTYH | NABP2 | NEIL1 | NEIL2 | NEIL3 | NHEJ1 | NTHL1 |
| NUDT1 | OGG1 | PARP1 | PARP2 | PARP3 | PARP4 | PCNA |
| PER1 | PMS1 | PNKP | POLB | POLD3 | POLD4 | POLE2 |
| POLE3 | POLE4 | POLG | POLH | RAD23A | RAD23B | RAD51C |
| POLI | POLK | POLL | POLM | POLN | POLQ | PPP4R1 |
| PPP4R2 | PPP4R4 | PRKDC | PRPF19 |  | RAD1 | RAD18 |
| RAD52 | RAD54B | RAD9A | RAD9B | RBBP8 | RBX1 | RDM1 |
| RECQL | RECQL4 | RECQL5 | REV1 | REV3L | RFC1 | RFC2 |
| RFC3 | RFC4 | RFC5 | RIF1 | RMI1 | RMI2 | RNF168 |
| RNF4 | RNF8 | RPA1 | RPA2 | RPA3 | RPA4 | RRM2B |
| SEM1 | SETMAR | SHPRH | SLX1A | SLX4 | SMUG1 | SPO11 |
| SPRTN | TDG | TDP1 | TDP2 | TELO2 | TOP3A | TOP3B |
| TOPBP1 | TP53BP1 | TREX1 | TREX2 | UBE2A | UBE2B | UBE2N |
| UBE2T | UBE2V2 | UNG | USP1 | UVSSA | WDR48 | WRN |
| XAB2 | XPA | XPC | XRCC1 | XRCC2 | XRCC3 | XRCC4 |
| XRCC5 | XRCC6 | ABCB11 | ABI1 | ACKR3 | ACSL3 | ACVR1 |
| ACVR1B | ACVR2A | AFF3 | AFF4 | AMER1 | ANK1 | APOBEC3B |
| AREG | ARHGAP5 | ARID1B | ARID2 | ARNT | ASXL1 | ATP1A1 |
| ATP2B3 | AXIN1 | AXIN2 | AXL | B2M | BAP1 | BAZ1A |
| BCL10 | BCL11A | BCL11B | BCL2 | BCL2L1 | BCL6 | BCOR |
| BCORL1 | BIRC3 | BIRC5 | BMP5 | BMPR1A | BRD4 | BTG1 |
| BUB1B | CACNA1D | CALR | CAMTA1 | CANT1 | CARD11 | CARS1 |
| CASP8 | CBFA2T3 | CBFB | CBL | CBLB | CCDC6 | CCNB1IP1 |
| CCND2 | CCNE1 | CCNO | CD79A | CD79B | CDC73 | CDH1 |
| CDH10 | CDH11 | CDK2 | CDK8 | CDKN1A | CDKN1C | CDKN2C |
| CDX2 | CEBPA | CENPX | CHD2 | CHD4 | CHIC2 | CIC |
| CIITA | CLIP1 | CLTCL1 | CNBP | CNOT3 | COL7A1 | CREB3L1 |
| CREB3L2 | CREBBP | CRKL | CRLF2 | CRNKL1 | CRTC1 | CRTC3 |
| CSF3R | CTCF | CTNND2 | CTR9 | CUL1 | CUX1 | CXCR4 |
| CYLD | CYP17A1 | CYP2C19 | CYSLTR2 | DAXX | DDIT3 | DDX10 |
| DDX3X | DDX5 | DDX6 | DICER1 | DIS3 | DIS3L2 | DKC1 |
| DNM2 | DNMT1 | DOCK8 | DPYD | DROSHA | EBF1 | EED |
| EIF3E | EIF4A2 | ELANE | ELF3 | ELF4 | ELK4 | ELL |
| ELOA | EMSY | EP300 | EPAS1 | EPHA7 | EPHB1 | EPS15 |
| ERC1 | EREG | ERF | ETNK1 | ETV6 | EWSR1 | EXT1 |
| EXT2 | EZR | FAH | FAM135B | FAM47C | FAS | FAT4 |
| FES | FH | FHIT | FOXL2 | FOXP1 | FRK | FUBP1 |
| FUS | G6PD | GALNT12 | GAS7 | GATA1 | GATA2 | GATA3 |
| GBA | GFI1 | GJB2 | GNA13 | GPC3 | GRB2 | GREM1 |
| GRIN2A | GSK3B | GSTT1 | H3-3A | H3C2 | HDAC1 | HEY1 |
| HFE | HIF1A | HIP1 | HLA-A | HLA-B | HLA-C | HMBS |
| HMGA2 | HNF1A | HNRNPA2B1 | HOOK3 | HOXA11 | HUS1B | IKBKE |
| IKZF1 | IL6ST | IRS2 | ITGAV | ITK | JMJD1C | JUN |
| KCNJ5 | KDM5A | KDM5C | KDM6A | KLF4 | KMT2A | KMT2C |
| KMT2D | KNL1 | LASP1 | LATS1 | LATS2 | LCK | LEF1 |
| LIFR | LMNA | LMO1 | LZTR1 | MAP2K4 | MAP3K1 | MAPK1 |
| MAX | MECOM | MED12 | MEF2B | MEN1 | MGA | MITF |
| MLLT3 | MLST8 | MPL | MTAP | MYB | MYCL | MYD88 |
| MYOD1 | NAB2 | NCOA3 | NCOR1 | NCOR2 | NDRG1 | NFE2L2 |
| NFIB | NHP2 | NME1 | NONO | NOP10 | NOTCH1 | NOTCH2 |
| NOTCH3 | NOTCH4 | NPM1 | NRG3 | NSD2 | NSD3 | NT5C2 |
| NUP93 | PAK1 | PAX3 | PAX5 | PAX7 | PAX8 | PDGFB |
| PDPK1 | PEDS1 | PER2 | PER3 | PHF6 | PHOX2B | PICALM |
| PIK3CB | PIK3CD | PIK3R3 | PIM1 | PLXNB1 | POT1 | POU2AF1 |
| POU5F1 | PPARG | PPM1D | PPP2R1A | PPP4R3A | PPP4R3B | PPP6C |
| PRCC | PRDM1 | PRDM16 | PRDM9 | PREX2 | PRF1 | PRKACA |
| PRKAR1A | PRKCH | PRSS1 | PSIP1 | PTK2 | PTK6 | PTPN11 |
| PTPN13 | PTPRD | PTPRT | QKI | RAD21 | RAD54L2 | RANBP2 |
| RAP1GDS1 | RASA1 | RBM10 | RFWD3 | RGS7 | RHBDF2 | RHEB |
| RHOA | RHOH | RIT1 | RNF213 | RPS6KA3 | RPS6KB1 | RUNX1 |
| RUNX1T1 | SBDS | SDC4 | SDHA | SDHAF2 | SDHB | SDHC |
| SDHD | SERPINA1 | SERPINB3 | SETBP1 | SF3B1 | SFPQ | SGK1 |
| SH2B3 | SH2D1A | SHOC2 | SLC25A13 | SLC29A1 | SLC34A2 | SLC45A3 |
| SLIT2 | SMAD2 | SMAD3 | SMAD4 | SMARCA1 | SMARCA2 | SOCS1 |
| SOS1 | SOX2 | SOX9 | SPEN | SPOP | SPRED1 | SPTA1 |
| SRGAP3 | SRSF2 | SRY | STAT3 | SUFU | SUZ12 | TBL1XR1 |
| TBX3 | TCF3 | TCF7L2 | TCL1A | TEAD2 | TET2 | TFE3 |
| TGFBR1 | TGFBR2 | THBS2 | TIMELESS | TMEM127 | TMPRSS2 | TNFAIP3 |
| TOP2A | TP63 | TPMT | TRAF7 | TRIM37 | TSHR | TSPAN31 |
| TYK2 | U2AF1 | UGT1A1 | UROD | USP6 | USP8 | WAS |
| WIF1 | WT1 | XPO1 | YAP1 | ZFHX3 | ZNF217 | ZNF479 |
| ZNF703 | ZNF750 | BCR |  |  |  |  |

**Supplementary Figure Legend**

**Supplementary Figure 1. CONSORT diagram. CONSORT diagram of 30 patients enrolled and samples analyzed**

**Supplementary Figure 2. Kaplan-Meier estimates of progression-free survival and overall survival based on treatment response in patients.**

**Supplementary Figure 3. The relationship between baseline maxVAF levels and treatment response**

**Supplementary Figure 4. Kaplan-Meier estimates of progression-free survival and overall survival based on changes in maxVAF after treatment in patients.**
